# Effects of exogenous melatonin on expressional differences of immune-related genes in cashmere goats

**DOI:** 10.3389/fgene.2022.967402

**Published:** 2022-10-24

**Authors:** Yuan Chai, Zaixia Liu, Shaoyin Fu, Bin Liu, Lili Guo, Lingli Dai, Yanyong Sun, Wenguang Zhang, Chun Li, Taodi Liu

**Affiliations:** ^1^ College of Agronomy, Animal Husbandry and Bioengineering, Xing’an Vocational and Technical College, Ulanhot, China; ^2^ College of Animal Science, Inner Mongolia Agricultural University, Hohhot, China; ^3^ Inner Mongolia Academy of Agricultural and Animal Husbandry Science, Hohhot, China; ^4^ Institute of ATCG, Nei Mongol Bio-Information, Hohhot, China; ^5^ Nei Mongol BioNew Technology Co., Ltd., Hohhot, China; ^6^ College of Animal Science and Veterinary Medicine, Tianjin Agricultural University, Tianjin, China; ^7^ Kunming Institute of Zoology, Chinese Academy of Sciences, Kunming, China; ^8^ College of Animal Science and Technology, Inner Mongolia University for Nationalities, Tongliao, China; ^9^ School of Basic Medical Sciences, Inner Mongolia Medical University, Hohhot, China

**Keywords:** cashmere goat, immune genes, melatonin, wool growth, high sulfur protein genes

## Abstract

The interplay between melatonin and immune system is well recognized in humans. The true integration of research on cashmere goat is still far from clear, especially for cashmere goat maintained in wool and cashmere growth. In this study, we applied various approaches to identify the complex regulated network between the immune-related genes and transcription factors (TFs) and to explore the relationship between melatonin and gene expression in cashmere goats. In total, 1,599 and 1756 immune-related genes were found in the blood and skin of cashmere goats, respectively, and 24 differentially expressed immune-related GO terms were highly expressed in blood after melatonin implantation. We studied the melatonin-dependent networks between the TFs and immune-related genes in cashmere goat. The 3 major regulatory networks were interconnected through TFs. The TFs, such as *PHF5A, REXO4, STRAP, JUNB, GATAD2A, ZNF710,* and *VDR*, were also expressed in the blood and skin tissue of cashmere goat. In addition, most genes in these networks, such as *VDR, JUNB,* and *Trib3*, were involved in WNT pathway, which is related to cashmere wool growth regulation*.* On the network basis, we developed a knockout mouse model to identify the network interaction. We observed that 8 high-sulfur protein genes, 12 keratin (KRT) genes, and 19 keratin associated protein (KRTAP) genes related to the growth of cashmere wool were almost not expressed in *Trib3* ^−/−^ rat skin. Our results suggested that the expression of genes related to wool and cashmere growth may be regulated by the interaction network between genes affected by melatonin and immune-related genes. In summary, we outlined some particularly promising ways for future research on immune-related genes of cashmere goats and the role of melatonin in wool and cashmere growth.

## Introduction

The wool and cashmere growth is regulated by several mechanisms. Overall, the hair follicle (HF) is one of the most important factors of wool and cashmere growth (Jiao et al., 2019; Pazzaglia et al., 2019; He et al., 2020; Nocelli et al., 2020). Biological implications of the molecular regulatory mechanisms underlying the periodic development of HFs are important for the future studies on cashmere wool growth. Decades ago, it was reported that melatonin and immune are involved in regulating hair growth and the periodicity of HFs (Fischer et al., 2010; Wang and Higgins, 2020).

The correlation between the immune and HF has been confirmed in previous studies (Paus et al., 1999). The testis, brain, adrenal cortex, and fetal placenta are some well-defined immune “privileged” tissue compartments in mammals, and HF is one of them. Paus et al. studied the immune privilege function of HFs during hair bulb growth and speculated that a constitutive active protection system can protect HFs from immune injury. System studies of the regulatory T-cells in skin revealed that they play a major role in HF biology by promoting the function of the stem cells of HF (Ali et al., 2017). Interestingly, immune cell types change and number involved in hair growth and dependent on remodeling all skin layers (Castellana et al., 2014; Joost et al., 2020). With the continuous progress in immune and HF research, the immune cells in various stages of HF cycle and the immune cells in diseases caused by normal HF interference are becoming more and more complex. For example, recruitment of immune cells to the developing HF contributes to the embryogenesis and the skin immune system changes with the hair cycle (Paus et al., 1998). Dendritic epidermal T-cells are distributed in the epithelium of HFs in mice and humans (Hashizume et al., 1994; Paus et al., 1994; Nagao et al., 2012), which act closely with basal keratinocyte stem cells and promote wound healing *via* fibroblast growth factor (Jameson et al., 2004).

A study reported that melatonin, a major neuroendocrine regulator, is crucial for the function of fur phenotype and photoperiod dependent environment. The study further addressed various aspects, including melatonin changing the yield of wool and cashmere, fur development and cycle frequency, seasonal molting, and fur color (Paterson et al., 1994; Kobayashi et al., 2005). Guerrero reported that the production of melatonin is related to the circadian rhythm and seasonal changes of the immune system, the impact of melatonin injection on the immune system, and the existence of melatonin receptors in the immune system (Guerrero and Reiter, 2002). However, the role of melatonin and immunity in HF biology is still not completely clear. Because of the complexity of molecular interaction and metabolism, the exact function of melatonin and immunity in wool and cashmere biology is still unclear.

In this study, we proposed a hypothesis that the regulation of villus growth is regulated by the interaction between immune-related genes and melatonin. We systematically analyzed the immune-related genes of cashmere goats to identify the important genes and potential factors in the regulatory network of villus growth. The present study revealed a complex immune landscape consisting of multiple regulation by melatonin and immune-related genes in the blood of cashmere goat.

## Materials and methods

### Ethics approval and consent to participate

#### Consent for publication

All samples were collected in accordance with the International Guiding Principles for Biomedical Research Involving Animals and approved by the Special Committee on Scientific Research and Academic Ethics of Inner Mongolia Agricultural University, responsible for the approval of biomedical research ethics of Inner Mongolia Agricultural University (Approval No. [2020] 056). We confirm that all methods were performed in accordance with relevant guidelines and regulations and the experimental procedure complied with the Animal Research Reporting of *In Vivo* Experiments (ARRIVE) guidelines.

### Experimental animals and sample collection

The data were obtained from the Genome Sequence Archive in BIG Data Center, Beijing Institute of Genomics (BIG), Chinese Academy of Sciences, including the RNA-seq data of 72 skin samples and 72 blood samples from 6 16-month-old female cashmere goats over 12 months. The accession number is CRA004598, which can be accessed from https://ngdc.cncb.ac.cn/gsa. Six cashmere goats were divided into two groups, including melatonin-implanted group (treatment group) and melatonin-nonimplanted group (control group), with 3 cashmere goats in each group (Chai et al., 2021). During the experiment, 2 mg/kg body weight melatonin was implanted subcutaneously behind the ears of the treatment group goats, whereas no melatonin was implanted in the control group. One month after implantation, scapula skin samples (size, 1 cm2) and venous blood samples were collected. Samples were quickly frozen in liquid nitrogen and were stored in a −80°C freezer (Chai et al., 2021).

The *Trib3* knockout (KO) mice were established by Dr. Taodi Liu. The adult male homozygous (−/−), heterozygous (+/−), and wild-type (+/+) Wistar rats were selected. Detailed genetic information of *Trib3* KO mice can be found in a previous article (Wang, 2020). The skin (size, 1 cm^2^), brain, ductus deferens, epididymics, kidney, liver, testis, and spleen tissues were collected, all 24 samples were quickly frozen in liquid nitrogen, and kept in a -80°C freezer.

### RNA-seq library construction and sequencing

For the RNA-seq library, total RNA was extracted from all rat tissue samples using TRIzol Reagent as per the manufacturer’s instructions. After the RNA samples were quantified, mRNAs were enriched with oligo (DT) magnetic beads. Further, the fragment buffer was added to break the mRNA into shorter fragments. Using mRNA as the template, single-stranded cDNA was synthesized using a randomized hexamer, followed by the synthesis of double-stranded cDNA by adding buffer, dNTPs, DNA polymerase I, and RNase H. Further, the double-stranded cDNA was purified using AMPure XP beads, and the second strand of cDNA containing U was degraded using USER enzyme. Purified double-stranded cDNA was repaired, followed by the addition of a tail and connection to the sequencing connector. Next, AMPure XP beads were used to select the fragment size. Finally, the final library was obtained *via* PCR amplification and purification. After RNA-seq library construction, Qubit 2.0 was used for preliminary quantification, followed by library insert size detection using Agilent 2,100. After the inserts met the expectations, qPCR was used to accurately quantify the effective concentration of the cDNA library and ensure its quality. In total, 24 libraries were constructed from the samples as the final sequencing library. High-throughput sequencing was performed using the illumina novaseq 6,000 platform with a paired-end sequencing.

### Quantification of the transcript levels and differentially expressed gene analysis

High quality; clean data of rats obtained by filtering data according to strict standards can be used for further research and publication (Supplementary Table S1). When the N content in any sequencing read is >10% of the base number of the read or when the low quality base number (Q ≤ 5) base present in any sequencing read was >50% of the base number of the read, the paired reads were removed. Further, we downloaded the rat genome (Rnor_6.0) and annotation files from the ensemble database. Hisat2 was used to align clean reads for each sample against the rat reference genome. StringTie (Pertea et al., 2015) program was used to quantify the transcript levels in rats.

We used Hisat2 (Kim et al., 2015) to match the read segments to the reference genome, which was obtained from the goat reference genome and its corresponding genome annotation file in the NCBI database (ARS1) (Dong et al., 2013). The differentially expressed genes (DEGs) were detected using limma in the R package (Ritchie et al., 2015) with default parameters to screen DEGs according to FPKM. The genes with |log_2_fc| > 1 and *p* < 0.05 were identified as DEGs.

### Construction of consensus weighted gene coexpression network and GCNs

The TFs with FPKM>1 that were expressed in at least two samples were kept, 686 TFs common in the control and treatment groups were retained. The expression data from 36 control and 36 treatment samples underwent consensus module analysis (Langfelder and Horvath, 2008) using the weight gene coexpression network analysis (WGCNA) package for R. The first step was to choose the soft thresholding power to which coexpression similarity was raised to calculate adjacency. Soft thresholding power was set to 12 and minimum module size to 30. Modules underwent hierarchical clustering, and similar modules were merged at a cut height of 0.25. The expressed TFs were identified from 4 modules that were highly conserved between the two datasets. Module eigengene was calculated for each module as the first principal component of gene expression for that module. Correlation analysis was performed to relate module eigengene to external traits including 71 immune-related DEGs.

The regulatory network between the 12,444 expressed genes (FPKM > 1 genes in at least 2 of the 72 samples) and 686 TFs was subjected to the gene coexpression network (GCN) (Chang et al., 2019) analysis for the identification and classification of gene coexpression network. FPKMs of all genes including TFs were combined and used for the calculation of the Pearson’s correlation coefficient (PCC) to predict potential gene regulatory networks. First, the PCC of the FPKM values was calculated for all pairs of 686 TF genes and 12,444 genes under the control and treatment groups, respectively. We obtained the positive and negative cutoff values with *p* < 0.05 for each condition in the Cutoff program (Supplementary Table S2). In the second step, we construct eight coexpression types of GCN under two conditions (C1 and C2): C1+C2+, C1+C20, C1+C2–, C10C2+ C1–C2+, C1–C2–, C1–C20, and C10C2–, where +, -, 0 represents the positive, negative, and no coexpression, respectively. Such as GCN in January, we defined PCC 0.85 and *p* < 0.05 as the criteria for the coexpression for TF and non-TF genes in control group. Therefore, the genes were defined as positively coexpressed (denoted as C1+ according to the dataset) if PCC was ≥0.85. Furthermore, the genes were identified asnoncoexpressed (denoted as C10) if −0.85 < PCC <0.85. If PCC was ≤-0.85, the genes were identified as negatively coexpressed (denoted as C1−). Considering the coexpression status under C1 and C2 together, if both genes were positively coexpressed under C1 and C2, they belonged to the set of C1+ C2+. Similarly, if they were positively coexpressed only under C1 (or C2), they belonged to C1 + C20 (or C10C2 +) and were not positively coexpressed under C2 (or C1).

### Weight gene coexpression network and expression-based genome-wide association studies analyses

In the blood control group, 5,497 genes (FPKM > 5 in all samples) were highly expressed. Therefore, a coexpression network was constructed using the WGCNA. The parameter β was given a default value from 1 to 30, as determined by the function SFT $powerestimate, with the minimum number of modules chosen as 30. The expression pattern of the module genes in each sample was represented by the module eigenvalue (ME). The MEs were calculated for the same expression pattern in the treatment group.

Further, the MEs of the control and treatment groups as the trait input data and eGWAS was conducted. The BAM file was used to detect single nucleotide polymorphisms (SNPs) using the samtools. SNPs were filtered using the following parameters: BIg -q 20 -d 1,000. The Binary Call Format (BCF) output file was redirected to the bcftools program to convert it to Variant Call Format (VCF) format. A total of 72 blood samples were genotyped for 7,919 (DP ≥ 10 in all samples) SNPs. To detect expression-associated eSNPs, the MEs of the control and treatment groups as the trait and expression-based genome-wide association studies (eGWAS) was conducted with 7,919 genome-wide SNPs.

### Enrichment analysis and biomarker genes selection using random forest

For the TFs and expressed genes at each month of a GCN, a corresponding set of coexpressed genes can be identified. The Gene Set Enrichment Analysis (GSEA) (Subramanian et al., 2005) was conducted with the background set of all genes in every month GCN. Only gene-sets that passed conservative significance thresholds (FDR < 25%) were selected for display in the Enrichment Map (Daniele et al., 2010). We presented a random forest-based classification model to predict a biomarker gene from immune-related genes (Statistics and Breiman, 2001). A total of 645 immune-related genes (n = 72) were used as input. For the random forest-based modelling, the discovery cohort was randomly split into a training (75%) and test (25%) sets. Our mRNA-based classifier demonstrated excellent performance in discriminating the control and treatment groups in the test. The area under the receiver operating characteristic curve (AUC) for the sensitivity analysis was 89% (Supplementary Figure S1).

## Results

### Transcriptome-wide identification, characterization, and expression profile of immune-related genes/pathways and transcription factors in cashmere goats

**FIGURE 1:**
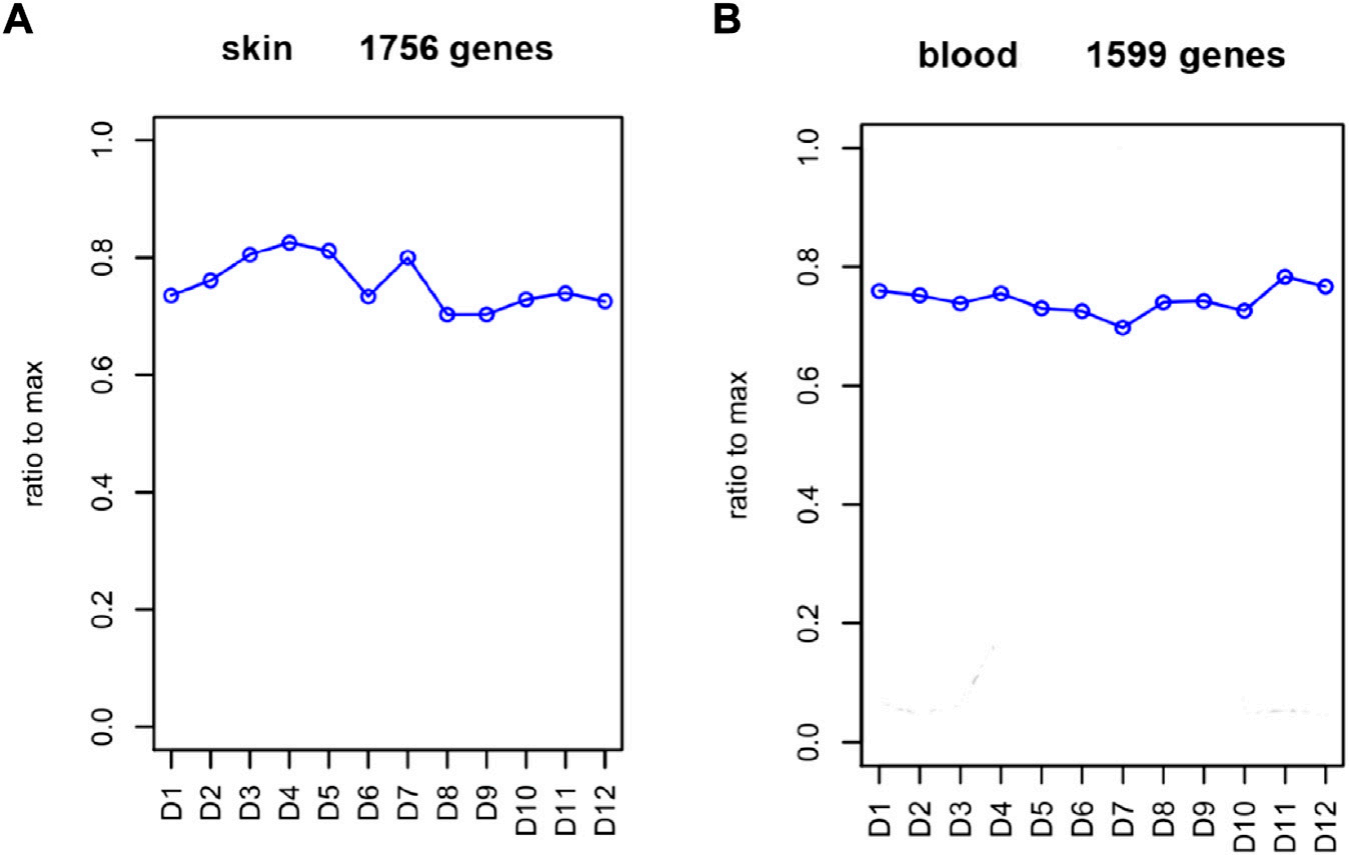
Abundance of immune-related genes in each month. **(A)** FPKM of immune-related genes in skin. **(B)** FPKM of simmune-related genes in blood. Abscissa represents month, and ordinate represents ratio to max. D represents the control group.

**FIGURE 2:**
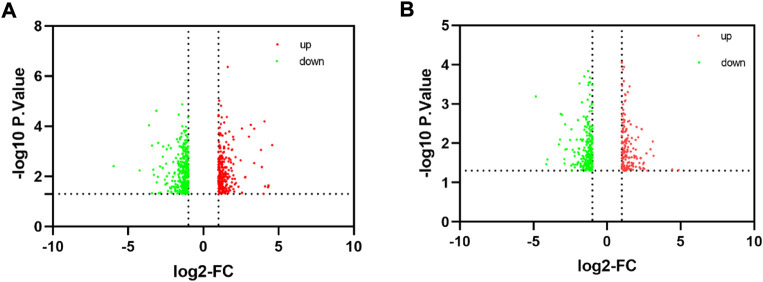
Identification of differentially expressed genes. **(A)** Volcano plot significantly differentially expressed genes in skin. **(B)** Volcano plot shows the distribution of differentially expressed genes in blood.

**FIGURE 3:**
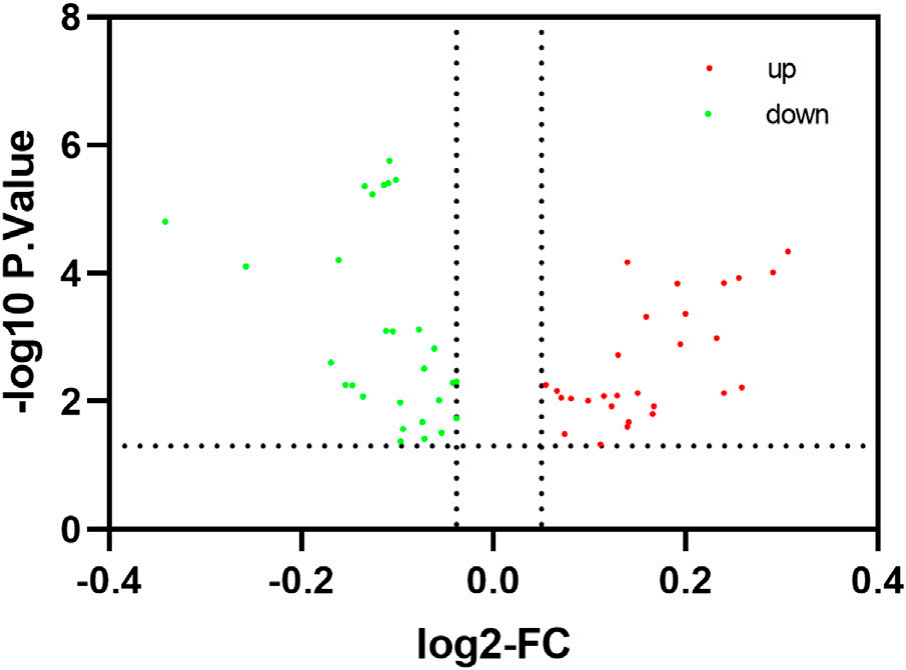
Identification of differentially expressed immune-related pathway. The red node indicates that the pathway is up-regulated in the treatment group of the blood, The green node indicates that the pathway is up-regulated in the control group of the blood.

To comprehensively examine the role of expression of immune-related genes during the HF development in the skin and blood tissues in the control and treatment groups, we collected the skin and blood samples at 12 continuous time points (from January to December). In total, 23,108 and 23,050 genes were identified in the skin of the control and treatment groups, respectively. Moreover, 21,378 and 21,304 genes were identified in the blood of the control and treatment groups, respectively. We defined a gene as expressed if FPKM was > 1 gene in at least 2 of the 72 samples. This resulted in 848 and 686 TFs being expressed in the skin and blood transcriptomes, respectively. Next, we searched all immune-related genes in cashmere goat genome from all annotated immune metabolic pathways in GO database (http://geneontology.org/). We found 3,308 immune related genes in 157 species covering 16,149 immune metabolic pathways, including 1,599 immune-related genes in cashmere goat blood. Furthermore, 1756 immune-related genes were found in cashmere goat skin samples. We observed that the immune-related genes expressed variably in the skin and blood over 1 year. The expression of immune-related genes in the skin demonstrated upward trends twice, from January to April and August to December, and a fluctuating trend was observed from May to July (Figure 1A). The trends of change in expression were not consistent in blood samples, with only January to July exhibiting a similar decreasing trend and August to December exhibiting an increasing trend (Figure 1B). The changes in gene expression in the skin and blood tissues associated with the treatment and control groups were analyzed. Overall, 680 DEGs were obtained in the skin (|log_2_fc| > 1, *p* < 0.05), with 366 and 314 genes upregulated in the control and treatment groups (Figure 2A). The number of upregulated DEGs in the blood was 160 and 247 in the control and treatment groups, respectively (Figure 2B). To determine the immune-related DEGs in the blood, 1,599 immune-related DEGs were analyzed. After screening, we obtained 71 immune-related DEGs in the blood of the control and treatment groups. A total of 18 genes were identified to be related to immune-related DEGs in the skin of the control and treatment groups. These cumulative changes in DEGs in the control and treatment groups between the blood and skin tissues may be minimal at the level of all genes. However, the concerned changes became evident as differential expression of two groups of immune-related genes in blood and skin. Our previous study reported that tissue-specific module genes involved in 687 pathways, such as adaptive immune response, innate immune response, immune system development, and regulation of leukocyte migration, were upregulated in the blood compared with skin (Chai et al., 2021). The functions involved immune-related GO terms were grouped for analysis in the blood. A total of 272 sets of immune-related GO terms were observed to be associated with 1,599 immune-related genes in the blood. Assessment of the expression of immune-related GO terms revealed 57 differentially expressed GO terms between the control and treatment groups in the blood of cashmere goats. In addition, 28 of the 47 differentially expressed immune-related GO terms were highly expressed in the treatment group (Figure 3).

### Identification of coexpression relationships and melatonin-dependent networks between the transcription factors and immune-related genes in cashmere goat

**FIGURE 4:**
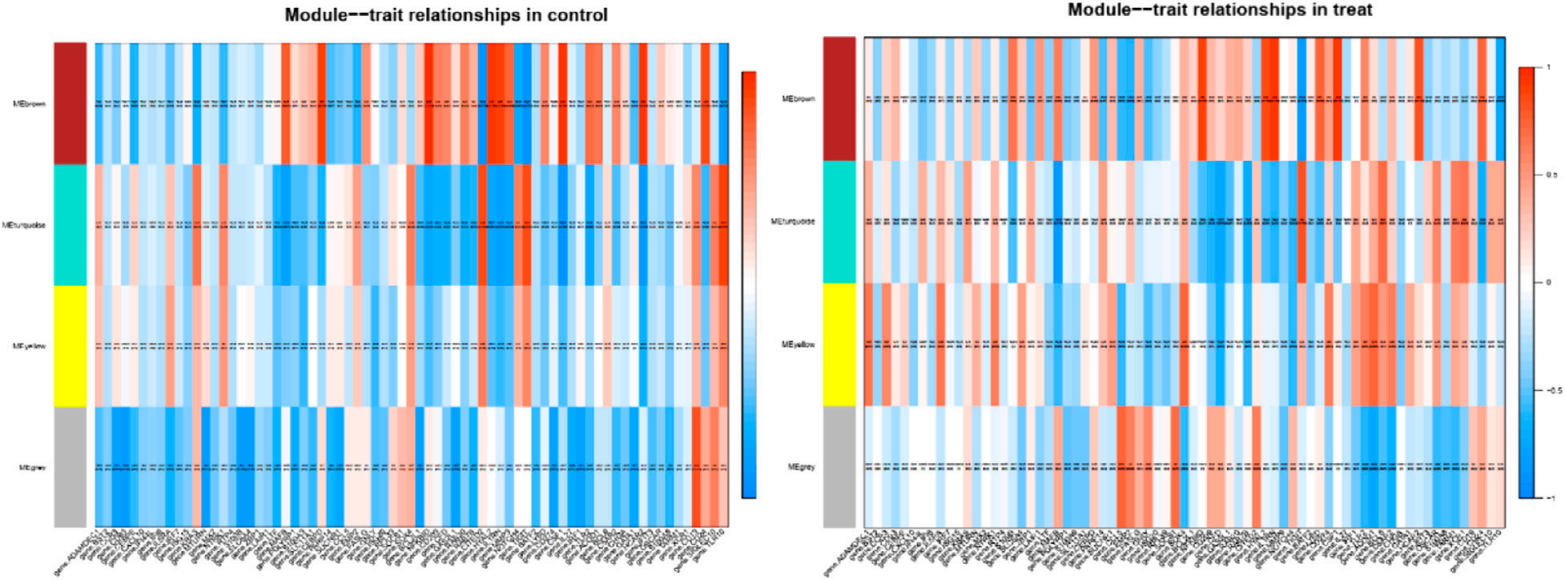
Module–differentially expressed immune-related genes association. Each row corresponds to a module, labeled with a color. Each column corresponds to differentially expressed immune-related genes. The color of each cell at the row-column intersection indicates the correlation coeffcient between the module and the tissue type.

Consensus module provides us with a method to assess whether gene modules co expressed under one condition are also strongly coexpressed under another condition. (Langfelder and Horvath, 2007; Langfelder et al., 2011). We performed a comprehensive analysis of coexpression relationships between the TFs and immune-related genes in the developmental stages across 1 year after melatonin implantation in blood. The TFs with FPKM > 1 that were expressed in at least two samples were kept, 686 TFs of common between the control and treatment groups were retained for the WGCNA. Coexpression networks were constructed on the same expression trends across the control and treat groups. The Consensus module is defined as a highly interconnected TFs cluster, and TFs in the same cluster have a high correlation coefficient. In this analysis, we identified 4 distinct consensus modules (labeled with different colors) shown in the dendrogram (Supplementary Figure S2). The 3 consensus modules (excluding grey module; the grey module contained unassigned TFs) correlated with 71 immune-related DEGs in the control and treatment groups (Figure 4). The brown module identified 118 TFs specific to the 10 immune-related genes in the control group and 6 immune-related genes in the treatment group. Gene coexpression turquoise modules were significantly associated with 11 immune-related genes in the control group and 8 immune-related genes in the treatment group (

**FIGURE 5:**
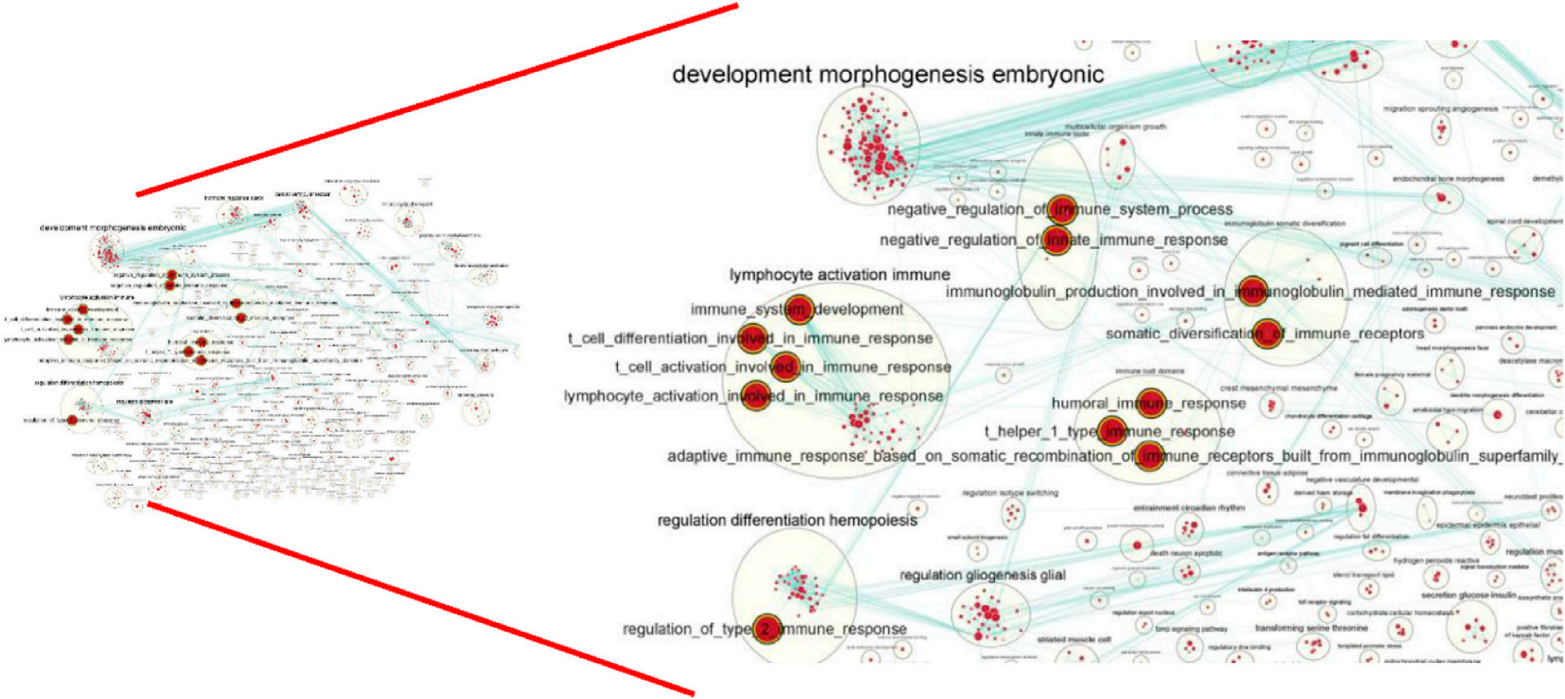
Global network of enrichment results of C10C2+ genes. The red indicate that the pathway is upregulated in the whole year except for May and December.

**FIGURE 6:**
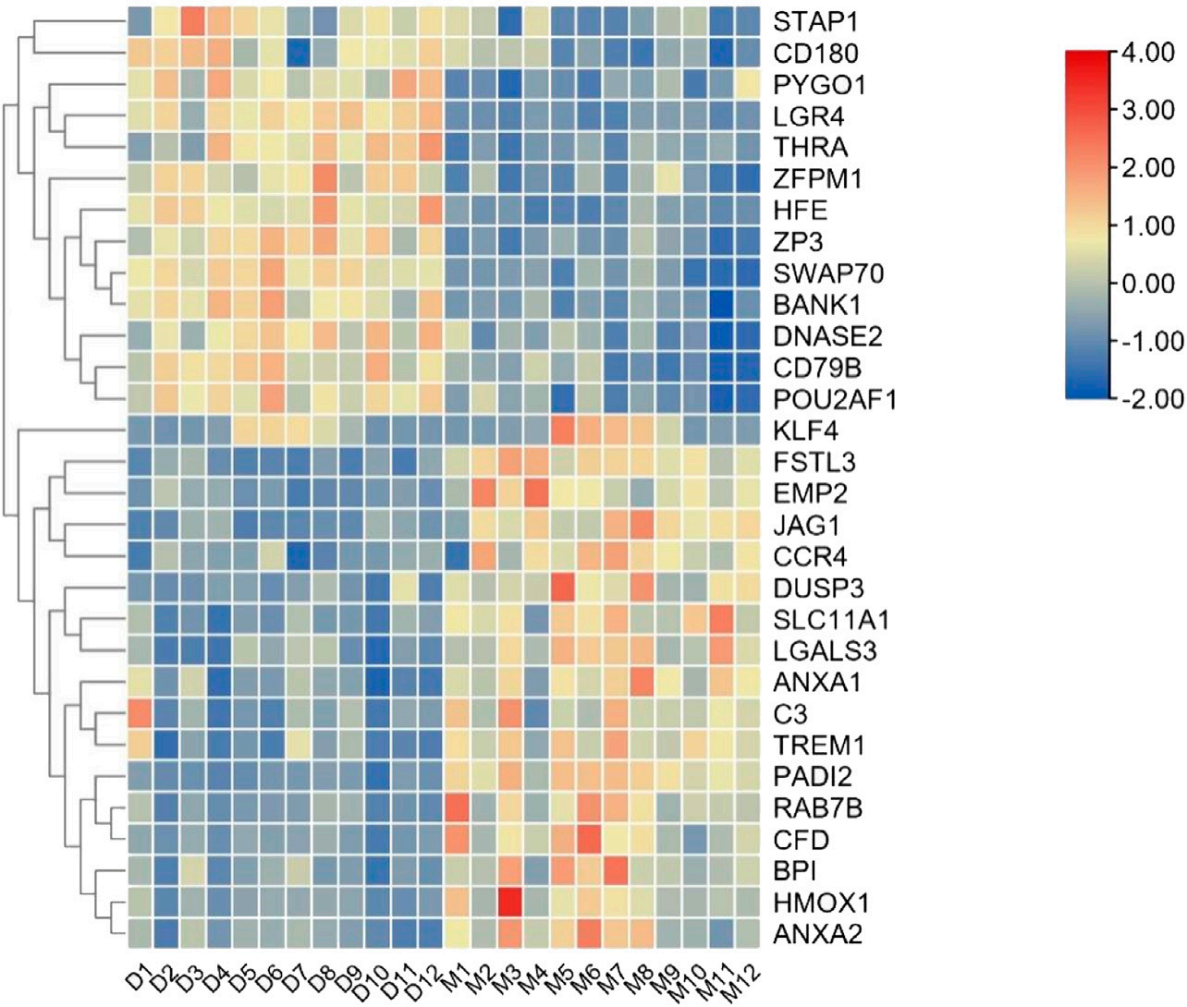
Expression pattern of 30 immune-related genes in blood. D is the control group, M is the implantation group, and the number is the month.

**FIGURE 7:**
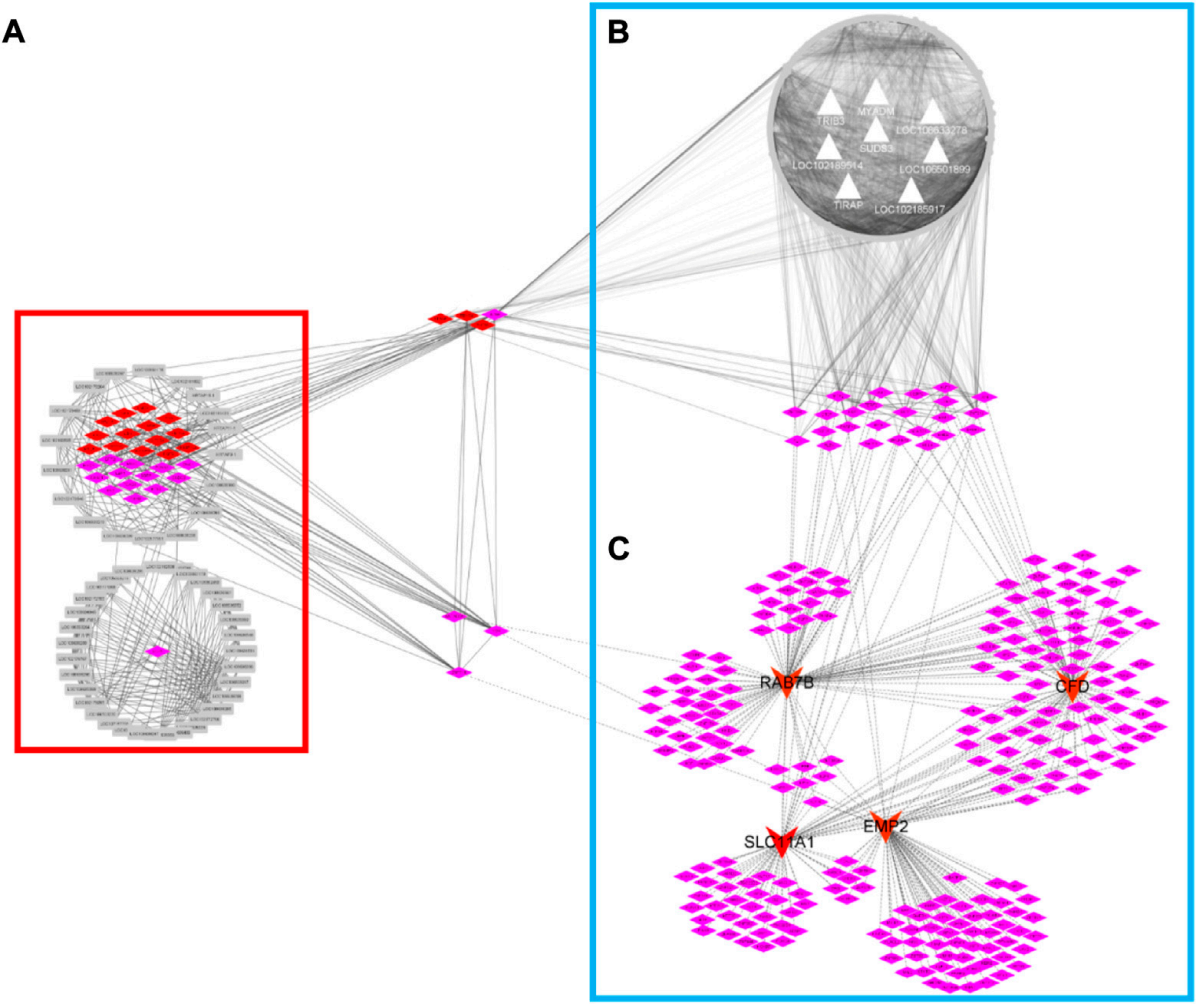
Co-expression regulatory network of high-sulfur protein gene, transcription factor, melatonin-dependent network and TIRB3-gene network. The red diamond node represents transcription factor, the pink node represents transcription factor cofactor. Gray node represents high sulfur protein gene in network **(A)**. The gray triangular node represents biomarker gene analysis by random forest machine learning approach in network **(B)**. The red arrow node represents biomarker gene of immune-related gene.

Table 1). This result prompted us to further identify whether the alteration in gene regulation networks is driven by melatonin and alteration of network is affected by the change in the regulatory relationship between TFs and genes. We used a comparative, GCN method to analyze the relationship between 686 TFs and 12,444 expressed genes (FPKM > 1 gene in at least 2 of the 72 samples) in blood. A total of 8 GCNs between the control and treatment groups were obtained. It is worth noting that for the C10C2- set, the coexpression relationships were dependent on melatonin (Supplementary Table S3) and independent of the months of May and December. The coexpression network between TFs and expressed genes was based on a PCC cutoff of 0.85 and *p* < 0.05. By identifying the functional categories among the coexpression genes and TFs at each month of the melatonin-dependent GCN, we reported 12 immune-related function sets containing 645 immune-related genes (Figure 5) using GSEA and Enrichment Map analysis. A total of 12 immune-related function sets were upregulated throughout the year except in May and December in the treatment group. A random forest machine learning approach was used for the analysis. In total, 30 immune-related genes were identified as the significant indicators for the control and treatment group (Figure 6). Based on random forest machine learning approach, the 30 immune-related genes assemblages that were indicative of high and low expressions were present throughout the sampling period. The 4 genes (immune-related genes such as *RAB7B*, *CFD*, *SLC11A1*, and *EMP2*) were differentially expressed in the treatment and control cashmere goats (|log_2_fc| > 1, *p* < 0.05). We explored and characterized the coexpression relationships and melatonin-dependent networks between the 249 TFs and 4 immune-related genes from the C10C2- set (Figure 7C).

**TABLE 1:** Module−trait relationships in control and treat.

Group	Module color	Pearson correlation coefficient	*p*-value	Gene name
control	brown	0.89	2e-05	*CEBPD*
		0.97	1e-10	*NFKBID*
		−0.9	9e-06	*CITED2*
		0.95	2e-08	*HYAL2*
		0.91	3e-06	*IL1RN*
		0.93	8e-07	*CSF1*
		0.93	7e-07	*HCAR2*
		0.86	1e-04	*CD24*
		−0.83	3e-04	*TLR10*
		−0.91	4e-06	*TIGIT*
	Turquoise	0.85	2e-04	*CITED2*
		0.84	2e-04	*TIGIT*
		0.91	3e-06	*TLR10*
		−0.89	2e-05	*CSF1*
		−0.85	1e-04	*HCAR2*
		0.9	1e-05	*NFKBID*
		0.92	2e-06	*IL1RN*
		0.93	6e-07	*CSF1*
		0.81	6e-04	*HCAR2*
		0.85	2e-04	*HYAL2*
		−0.84	2e-04	*TIGIT*
treat	brown	0.9	1e-05	*NFKBID*
		0.92	2e-06	*IL1RN*
		0.93	6e-07	*CSF1*
		0.81	6e-04	*HCAR2*
		0.85	2e-04	*HYAL2*
		−0.84	2e-04	*TIGIT*
	Turquoise	0.8	8e-04	*TIGIT*
		−0.88	4e-05	*MAFB*

### Integration of gene coexpression networks and expression-based genome-wide association studies analyses highlighted the candidate regulators implicated in melatonin-dependent immune gene interaction networks

To identify the regulatory mechanisms underlying the induction of the melatonin-dependent networks by immune-related genes, we performed eGWAS and WGCNA in blood samples. First, we used WGCNA for describing the correlation patterns among 5,497 genes (FPKM > 5 in all samples) in the control group. Using WGCNA, we identified 6 gene coexpression modules in the control group (Supplementary Figure S3). The number of genes in each module ranged from 6 (module grey) to 1,485 (module turquoise). Assessment of MEs for the 6 gene modules in the treatment group revealed that a great variation existed in the MEs compared with the control group. Further, the MEs of the control and treatment groups as the WGCNA trait input data and eGWAS was conducted with 7,919 genome-wide SNPs. Using eGWAS, the identified 25 eSNPs were mapped in intergenic regions andassociated with 20 genes in the control and treatment groups (Supplementary Table S4). Remarkably, we observed a significant genetic variation in the treatment group, and brown module associated genes are the most. Exploratory analyses were conducted with brown module genes and 9 biomarker genes. The assessment of regulatory network between brown module genes and biomarker genes was conducted based on the PCC in the treatment group. Regulatory gene networks (cor ≥ 0.8, *p* ≤ 0.05) were identified involving 991 genes and 8 biomarker genes, such as *LOC102189514*, *LOC106501899*, *LOC102185917*, *MYADM*, *LOC108633278*, *Trib3*, *TIRAP*, and *SUDS3* (Figure 7B)*.* The genes in the interaction network between brown module genes and biomarker genes seem to be closely related to RNA transport, RNA degradation, spliceosome, protein export, protein processing in the endoplasmic reticulum KEGG pathway, and gene enrichment in the regulatory network (Supplementary Figure S4). Because the expression of many genes in the genome is regulated by TFs, we observed that the network included 24 TFs for target gene sets in the treatment group, and these 24 TFs simultaneously regulated the melatonin-dependent networks between the TFs and immune-related genes in cashmere goats. We previously reported that high-sulfur gene expression is important for wool growth regulation (Chai et al., 2021). A total of 47 high-sulfur protein genes exhibited an interaction with TFs and cofactors with ASE in the skin of cashmere goat. These TFs and cofactors were inhibited after melatonin implantation. Our data are particularly alarming because overlapping TFs, such as *PHF5A*, *REXO4*, *STRAP*, *JUNB, GATAD2A*, *ZNF710*, *and VDR*, were already observed in 3 networks (Figure 7C), and these TFs were also expressed in blood and skin tissues of cashmere goat. This indicated that many biological functions cannot be explained by the function of a single gene or protein. Instead, they are the result of a network of interactions between two genes or between proteins and other molecules.

### Role of Trib3 in the regulation of the expression of high-sulfur protein genes in the rat skin

**FIGURE 8:**
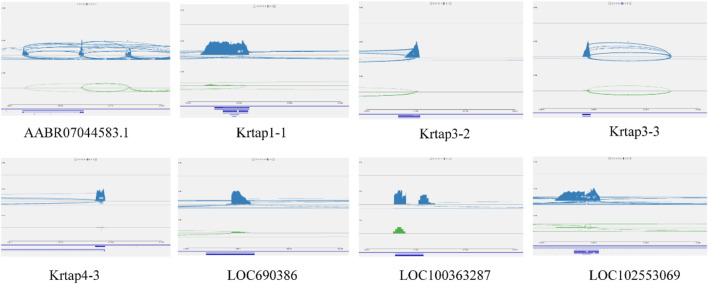
The high sulfur protein genes expression in more detail by IGV. The high sulfur protein gene were not expressed in adult male homozygous (−/−) skin tissue, the expression of high sulfur protein genes in the wild-type (+/+) Wistar rat skin was marked blue. The green node represent the expression of high sulfur protein genes in heterozygous (+/−) rat.

The identification and understanding of the gene regulatory network of wool growth regulation process after melatonin implantation is one of the main challenges. Another challenge is to identify the main driving factors and major regulatory genes that control the wool growth regulation. In this study, we observed that 3 major regulatory networks were interconnected through TFs, and some TFs, such as *PHF5A*, *REXO4*, *STRAP*, *JUNB*, *GATAD2A*, *ZNF710*, *and VDR*, were also expressed in the blood and skin tissue of cashmere goat. In addition, most genes in the network, such as *VDR*, *JUNB*, *and Trib3*, were involved in WNT pathway, which is related to wool growth regulation. The close cooperation between 3 networks gives rise to an interesting question: how does regulatory network collaborate with genes for controlling gene expression in skin and blood. To address this question, the network of *Trib3* gene in blood was selected as candidate genes because of the involvement of this gene in the WNT pathway. In the network of *Trib3* genes, the TFs, namely, *PHF5A*, *REXO4*, *STRAP*, and *JUNB* interact with the regulatory network of *Trib3* in blood and with high-sulfur protein genes in skin. Based on this, we guessed that the regulatory network of high-sulfur protein gene and *Trib3* gene also interacted in the skin. However, this is just a conjecture. Further, we investigated the impact of *Trib3* gene knockout on the high-sulfur protein genes expression in skin, brain, ductus deferens, epididymics, kidney, liver, testis, and spleen tissues in rat. Using homologous gene sequences of high-sulfur protein genes in cashmere goat as our query terms, we searched for 8 candidate genes from the rat using BLAST. We used the “absolute” standard for filtering, which simply removes BLAST hits that do not meet a top1 criterion; various possible criteria include bit score, E-value, and alignment length. The RNA-seq analysis revealed specific expression of 8 homologous genes (Supplementary Table S5) of high-sulfur protein genes in rat skin tissue (Supplementary Table S6). We investigated the expression of high-sulfur protein genes in more detail using IGV (Figure 8). The 8 high-sulfur protein genes, 12 KRT genes, and 19 KRTAP genes were not expressed in adult male homozygous (−/−) skin tissue. The expression of high-sulfur protein genes in the wild-type (+/+) Wistar rat skin was significantly increased. However, we observed that the decreased expression became conspicuous in adult male heterozygous (+/−) rat (Supplementary Table S7).

## Discussion

It is increasingly clear that the immune and melatonin might contribute to HF development. However, comprehensive analysis of the differentially expressed profiles of immune-related genes after implantation of melatonin and potential interactions between TFs and immune-related genes in blood and skin through integrating RNA-seq and construction of a rat gene knockout model are not available.

Researchers have confirmed that TFs, such as Lhx2 and csdc2, regulate the secondary HF cycle of cashmere goats, which are potentially important TFs in the hair growth cycle. In the present study, we obtained complete datasets of immune-related genes and TFs in cashmere goat after analyzing datasets from GO and Animal TFDB database. In addition, to further understand the molecular mechanism of immunity in the regulation of wool growth in cashmere goats, we analyzed the expression patterns of immune-related genes in blood and skin of cashmere goats.

Focusing on interaction between the TFs and immune-related genes, we identified melatonin-dependent gene regulatory networks that might serve as a basis for investigating mechanisms related to wool and cashmere growth function. Using GCNs and random forest machine learning approach, we identified 4 biomarker immune-related genes that were differentially expressed in the melatonin-implanted and control cashmere goats (|log_2_fc| > 1, *p* < 0.05), including *RAB7B*, *CFD*, *SLC11A1*, and *EMP2.* A recent study regarding the influence of the senescent cells on matrix production reported that the secretion of *CFD* promotes degradation of nearby fibroblasts in the dermal layer (Ezure et al., 2019). Evidence supports that *SLC11A1*, encoding a transport protein, is expressed in macrophage phagosome membranes, contributing to the ion transport (Vidal et al., 1993; Govoni et al., 1997; Canonne-Hergaux et al., 1999). Codon 169 of SLC11A1 gene encodes Gly in the R allele and ASP in the S allele. The two alleles are related to the sensitivity and drug resistance in the enteric typhoid infection by *Salmonella* (Malo and Skamene, 1994; Cellier et al., 1995; Vidal et al., 1995; Cellier et al., 1996; Cellier et al., 1997). The mutation of S allele can change the ion concentrations in the phagosome (Hackam et al., 1998; Zaharik et al., 2004). *SLC11A*1 gene is critical to regulating macrophage activation, oxidative and nitrosamine bursts (Fritsche et al., 2003), and expression of *TNF-a*, *IFN-c*, *IL-*1 (Lalmanach et al., 2001), and *MHC* II (Wojciechowski et al., 1999). *RAB7B* is a paralogous gene of *RAB7A* and is involved in membrane tracking in the late endosomal/lysosomal pathway (Osińska et al., 2011). A study reported that *EMP*2 may also be involved in cell proliferation and cell–cell interactions (Wadehra et al., 2003). In summary, although there is overwhelming information demonstrating the relationship between the immune and melatonin, many questions related to TFs and the mechanisms of other aspects are unanswered. We suspect that many additional mechanisms underlying the formation and maintenance of HFs and growth of wool and cashmere have not yet been discovered. We only see the “tip of the iceberg.”

Although SNPs found in RNA sequence data have limitations, the results are encouraging. Remarkably, we observed a significant genetic variation in the treatment group. The regulatory network between 991 genes and 8 biomarker genes, such as *LOC10218951*, *LOC10650189*, *LOC102185917*, *MYADM*, *LOC108633278*, *Trib3*, *TIRAP*, and *SUDS3*, was analyzed based on the PCC in the treatment group (Figure 7B). We observed that this network includes 24 TFs for target gene sets in the treatment group, and these 24 TFs simultaneously regulated the melatonin-dependent networks (Figure 7C) between the TFs and immune-related genes in cashmere goats. In our previous study, 47 high-sulfur protein genes exhibited interaction with TFs and cofactors with ASE in the skin of cashmere goat (Chai et al., 2021). These TFs and cofactors were inhibited after melatonin implantation in the skin of cashmere goat, and 13 genes in the interaction network were functionally associated with the function of hair growth and WNT pathway (Chai et al., 2021). Our data are particularly alarming because overlapping TFs, such as *PHF5A*, *REXO4*, *STRAP*, *JUNB*, *GATAD2A*, *ZNF710*, and *VDR*, were already observed in 3 networks (Figure 7) and were also expressed in blood and skin tissue of cashmere goat. In the study by Wang et al., *VDR* and *JUNB* gene specific expression at embryonic day 120 are thought to play an important role in HF and WNT pathway (Wang et al., 2020). The transcriptional activity of β-catenin-TCF4 was determined by Trib3 and β-catenin synergism, which then affects the initiation and progression of CRC (Hua et al., 2018). Therefore, *Trib3* was selected as the candidate gene. The major types of networks between the immune-related genes, high-sulfur protein genes, and TFs in blood and skin after melatonin implantation were constructed. The network contained 7 bridge genes in the cashmere goat of blood and skin. To investigate whether the network gene in blood affects the high-sulfur protein genes in skin, we analyzed the interaction between *Trib3* and high-sulfur protein in the skin (size, 1 cm^2^), brain, ductus deferens, epididymis, kidney, liver, testis, and spleen tissues of rats with *Trib3* gene knockout. Notably, 8 high-sulfur protein, KRT, and KRTAP genes were specifically expressed (Supplementary Table S6) in the rat skin tissue. These genes were not expressed in adult male homozygous (−/−) skin tissue, and the expression of high-sulfur protein genes in the wild-type (+/+) Wistar rat skin was significantly increased. However, we observed that the decreased expression became conspicuous in adult male heterozygous (+/−) rats.

Mature wool mainly consists of three layers: cortical, cuticle, and wool central core and medulla (Powell and Rogers, 1997a). Studies have reported that cortical keratin consists of microfibers (keratin intermediate filaments) of 8-nm diameter embedded in the matrix. Keratin-related proteins were located the matrix between microfibers (Plowman, 2003). A classification system was proposed based on whether the wool fiber contains keratin intermediate filaments (KIF). KRTm. nxpL is used to classify intermediate filaments, whereas other proteins with high-sulfur, ultra-high-sulfur and high glycine–tyrosine are used as keratin-associated proteins (abbreviated as KAPm.nxpL) (Rogers and Powell, 1993; Powell and Rogers, 1997b). Overall, 90% of KIF and KAP constitute the main body of wool fibers, and the spatial structure of KIFs and chemical bond of KAP in the matrix are believed to largely determine the physical properties of fibers (Gong et al., 2016). While discussing these aspects, the importance of their expression for cashmere fiber and length traits was emphasized, that is, through the current understanding of the mechanism by which keratin and keratin-associated proteins affect wool traits and the expression of specific genes as part of these mechanisms.

Compared with other species, in goats, keratin-associated protein and keratin can also be classified into its gene families based on the similarity of their amino acid sequences. Sequence analyses suggest that the KAP and KRT sequences identified to date probably represent different family members. The KAP1 family of goat fiber proteins, such as KAP1.1 and KAP1.4, are primarily described in recent years, which is a HS-KAP family (Zhang et al., 2011; Shah et al., 2013). Liu et al. used PCR-SSCP method to reveal that the KAP8.2 gene may be a potential molecular marker for determining the cashmere fiber diameter of cashmere goats, and the genotype polymorphism has no effect on the length of cashmere (Liu et al., 2007). KAP8.2 is strongly expressed in the cortical tissue of primary and secondary HFs of embryonic and adult goat skin with high homology at the nucleotide and amino acid levels in goats of different breeds and from different regions (Hang et al., 2008). The study on KAP6-1.2 in goats indicated that this gene was strongly located on the cortical layers of primary and secondary HFs in the skin of embryonic and adult goats (Zhang et al., 2007). The study by JIN et al. reported that KAP7.1 genes are specifically expressed in the HF, which can effectively regulate the HF growth and development in cashmere goats and may even affect the wool fiber diameter; the expression of secondary follicles was significantly higher in primary HFs (Jin et al., 2011). KAP8.2 is strongly expressed in the cortical tissue of primary and secondary HFs of embryonic and adult goat skin with high homology at the nucleotide and amino acid levels in goats of different breeds and from different regions (Zhang et al., 2008). KAP24-1 is one of the most recently discovered high-sulfur KAP gene in goats. Wang reported that this KAP gene domain was on goat chromosome 1 (Wang et al., 2019). Prior to the publication of Wang, KAP13.1 gene, were reported (Fang et al., 2005). The discovery of the polymorphisms of goat KAP13.1 genes has suggested that polymorphic variants might be relevant in determining fiber diameter. Not fully unexpectedly, the patterns of KAP11-1 expression in the caprine skin, to a certain degree, that of fiber diameter size. Real-time PCR results revealed that KAP11.1 exhibited higher expression in the catagen than in the anagen phase in the primary HFs. However, in the secondary HFs, KAP11.1 exhibited a significantly higher expression in the anagen than in the catagen phase. Moreover, KAP11.1 gene exhibited a strong expression in the inner root sheath and hair matrix and a lower expression in the hair bulb (Jin et al., 2017). A search for similar sequences in the caprine genome using the human KAP20-2 gene (KRTAP20-2) revealed a homologous sequence on chromosome 1, which contains an open reading frame of 189 bp, and the nucleotide homology with human KAP20-2 is 75% (Wang et al., 2017).

Even in the likely event that the new focus in immune-related gene research on the still insufficiently explored potential regulated genes of the network that we strongly advocate here. However, this focus is guaranteed to produce a rich immunological harvest in terms of improving our general understanding of the comprehensive regulation of wool and cashmere growth associated with melatonin, immune-related genes, high-sulfur protein genes, ASE, and SNPs.

## Data Availability

The datasets analyzed for this study can be found in the Genome Sequence Archive in BIG Data Center, Beijing Institute of Genomics (BIG), Chinese Academy of Sciences, under accession number CRA007385 and accession number CRA004598 that are publicly accessible at https://ngdc.cncb.ac.cn/gsa.

## Supplementary material

Supplementary Figure S1 ROC curve discrimination between the control and treatment group.

Supplementary Figure S2 Consensus weighted gene coexporession network analysis. Modules were identified in the resulting dendrogram using the Dynamic Tree Cut algorithm. A total of 4 modules were identified. Modules were distinguished from each other by assigning different colors.

Supplementary Figure S3 Network heatmap plot, all genes.

Supplementary Figure S4 “Genes of the regulatory networks target-biological process-signaling pathway” network.

Supplementary Table S1 Summary of RNA-sequencing results and quality data output.

Supplementary Table S2 The positive and negative cutoff values for the control group and the melatonin-implanted group.

Supplementary Table S3 Numbers of co-expression relationships in each developmental stage and GCN.

Supplementary Table S4 The module-associated SNP by eGWAS analysis.

Supplementary Table S5 The blast search results.

Supplementary Table S6 The expression of 8 high sulfur protein gene in rat tissues.

Supplementary Table S7 The expression of 12 KRT gene and 19 KRTAP gene in rat skin tissue.

## Abbreviations

HF, hair follicle; DEGs, differentially expressed genes; TFs, transcription factor; WGCNA, weight gene co-expression network analysis; GCN, gene co-expression network; PCC, Pearson correlation coefficient; ME, module eigenvalue; SNP, single nucleotide polymorphisms; eGWAS, Expression-based genome-wide association studie; AUC, receiver operating characteristic curve.
