# Supplementary figures and images for: Effects of exogenous melatonin on expressional differences of immune-related genes in cashmere goats
Source: Front Genet. 2022 Oct 24;13:967402. doi: 10.3389/fgene.2022.967402 (PMC9638969; doi:10.3389/fgene.2022.967402)

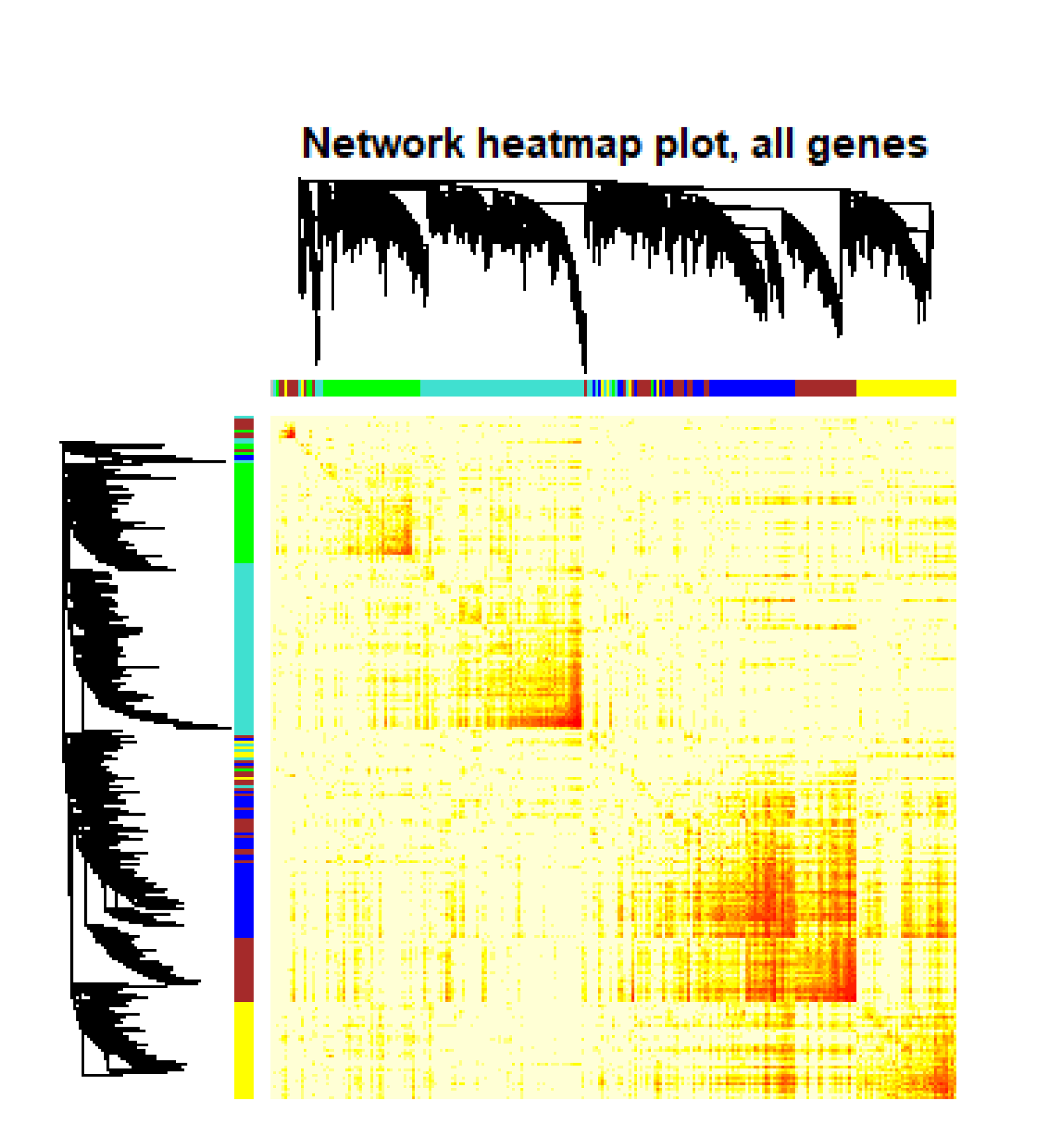

Supplement: Supplementary file 3 [file Image3.tif]

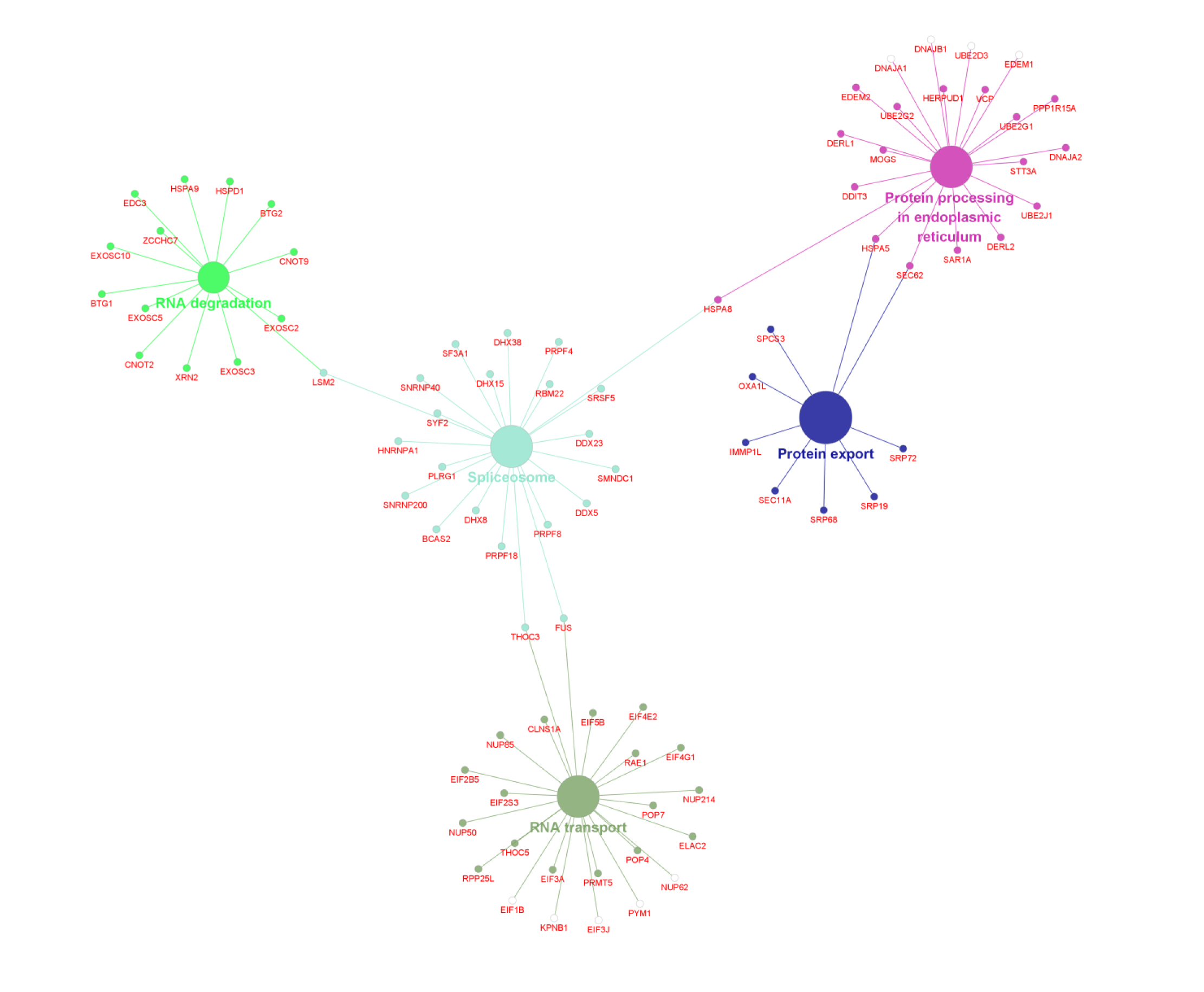

Supplement: Supplementary file 4 [file Image4.tif]

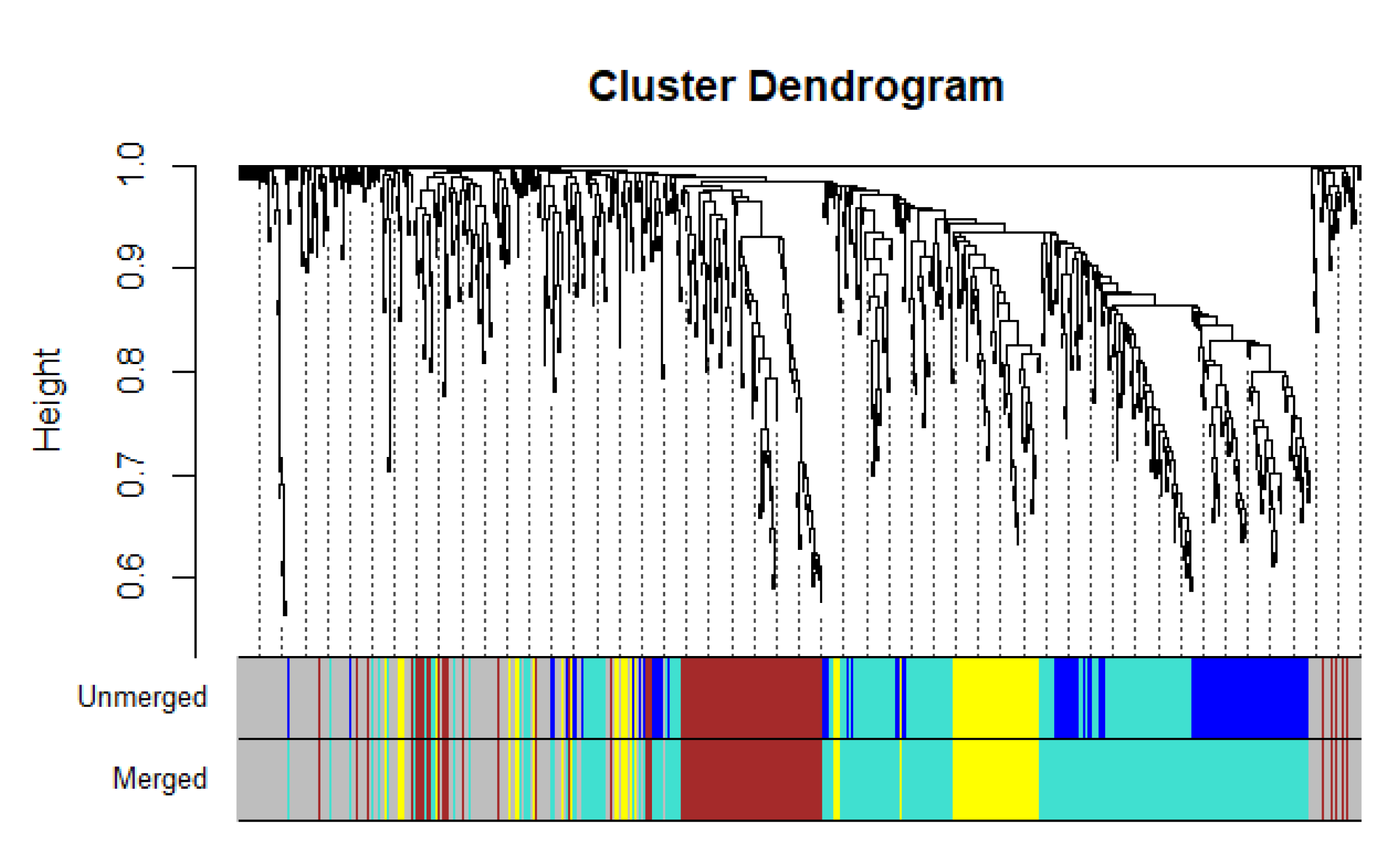

Supplement: Supplementary file 5 [file Image2.tif]

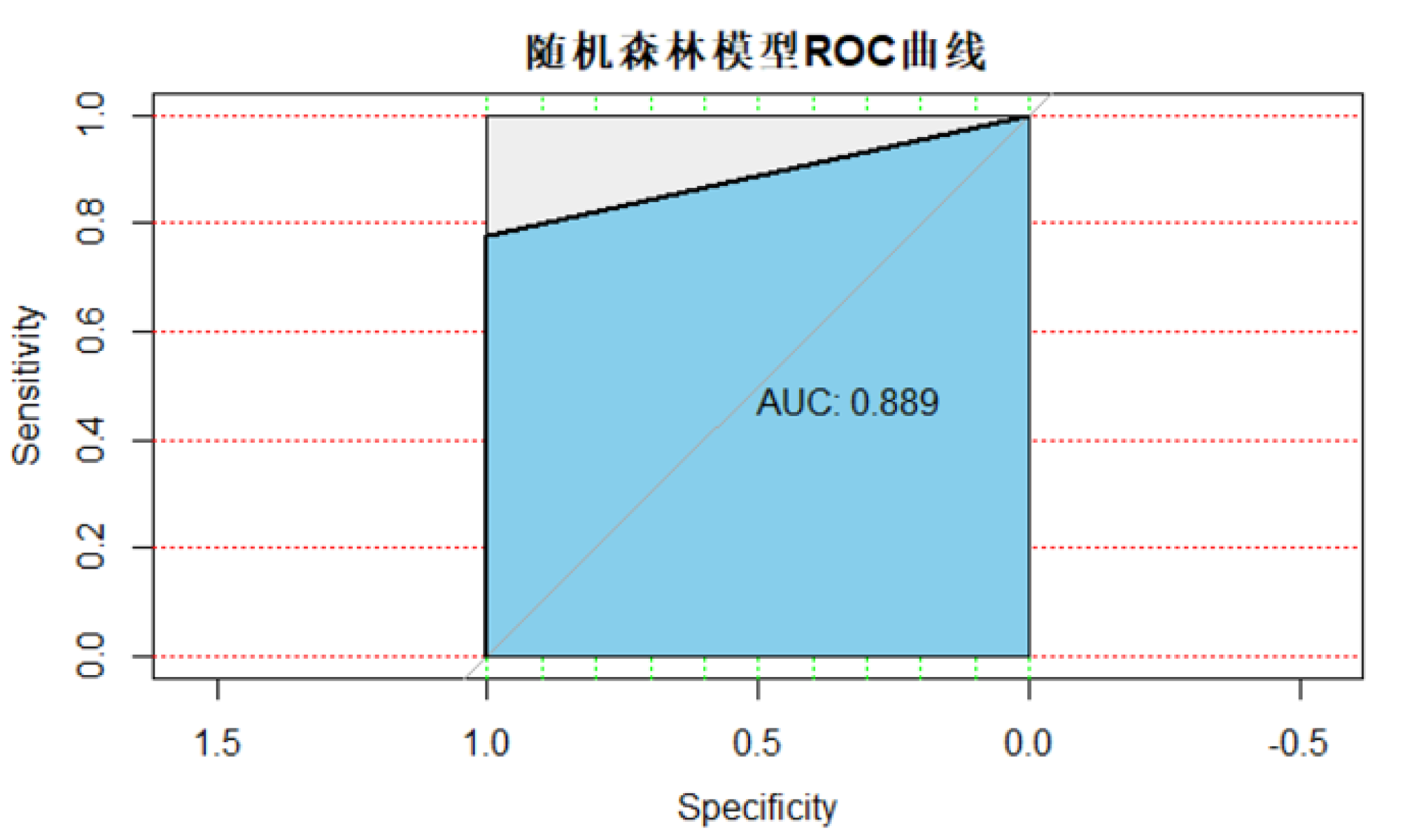

Supplement: Supplementary file 6 [file Image1.tif]
